# Effect of Metabolic Syndrome on Parkinson's Disease: A Systematic Review

**DOI:** 10.6061/clinics/2021/e3379

**Published:** 2021-12-01

**Authors:** Ana Patrícia da Silva Souza, Waleska Maria Almeida Barros, José Maurício Lucas Silva, Mariluce Rodrigues Marques Silva, Ana Beatriz Januário Silva, Matheus Santos de Sousa Fernandes, Maria Eduarda Rodrigues Alves dos Santos, Mayara Luclécia da Silva, Taciane Silva do Carmo, Roberta Karlize Pereira Silva, Karollainy Gomes da Silva, Sandra Lopes de Souza, Viviane de Oliveira Nogueira Souza

**Affiliations:** I Programa de Pos-graduacao em Neuropsiquiatria e Ciencias do Comportamento, Centro de Ciencias da Saude, Universidade Federal de Pernambuco, Recife, PE, BR.; II Departamento de Fisioterapia, Centro de Ciencias da Saude, Centro Universitario Osman Lins (UNIFACOL), Vitoria de Santo Antao, PE, BR.; III Centro Integrado de Tecnologias em Neurociencia (CITENC), Centro Universitario Osman Lins (UNIFACOL), Vitoria de Santo Antao, PE, BR.; IV Nucleo de Nutricao, Centro Academico de Vitoria (CAV), Universidade Federal de Pernambuco, Vitoria de Santo Antao, PE, BR.

**Keywords:** Metabolic Syndrome, Parkinson Disease, Vascular Risk Factors

## Abstract

Evidence shows that metabolic syndrome (MS) is associated with a greater risk of developing Parkinson's disease (PD) because of the increase in oxidative stress levels along with other factors such as neuroinflammation and mitochondrial dysfunction. However, because some studies have reported that MS is associated with a lower risk of PD, the relationship between MS and PD should be investigated. This study aimed to investigate the effect of MS on PD. Two authors searched five electronic databases, namely, MEDLINE, PubMed, Scopus, PsycINFO, Web of Science, and Science Direct, for relevant articles between September and October 2020. After screening the title and abstract of all articles, 34 articles were selected for full-text review. Finally, 11 articles meeting the eligibility criteria were included in the study. The quality of articles was critically evaluated using the Joanna Briggs Institute. Overall, we evaluated data from 23,586,349 individuals (including healthy individuals, with MS and PD) aged 30 years or more. In cohort studies, the follow-up period varied between 2 and 30 years. MS contributed considerably to the increase in the incidence of PD. In addition, obesity, a component of MS, alone can increase the probability of developing neurodegenerative diseases. However, despite few studies on MS and PD, changes in cognitive function and more rapid progression of PD disease has been documented in patients with MS using methods commonly used in research.

## INTRODUCTION

A quarter of the global population is estimated to be affected by metabolic syndrome (MS) (1). The prevalence of MS increases with age. In the United States of America, the prevalence of MS is 20%-25% in adults and 0.6%-13% in university students (2,3). A study involving 1125 nursing professionals of both sexes (average age: 37.1 years) who worked in primary health care in Brazil reported that the prevalence of MS is 23.7% in women and 29.4% in men (4).

The pathogenesis of MS is triggered some factors such as oxidative stress, which attacks cellular macromolecules such as proteins, lipids, and nucleic acids and promotes cellular dysfunction (5). Evidence shows that MS is associated with a higher risk of developing Parkinson's disease (PD) (6,7) because of the increase in oxidative stress as well as other factors such as neuroinflammation and mitochondrial dysfunction (8). However, because some studies have reported that MS is associated with a lower risk of PD, the relationship between MS and PD should be investigated (9).

Studies have indicated that the incidence of PD increases with age. The number of PD cases in the world is expected to double by 2030 (10). PD is the second most common neurodegenerative disease, with a prevalence of 0.3% in the general population, 1% in individuals aged >60 years, and 3% in individuals aged >80 years (11). PD affects central and peripheral neurons, including nigrostriatal dopaminergic neurons that coordinate motor function (12). However, pathological analyses have revealed that 40%-60% of dopaminergic neurons are lost before the onset of motor symptoms (13). Consequently, PD is clinically diagnosed at advanced stage. This non-symptomatic period may vary from 5 to >20 years and is called the prodromal phase of PD (14).

Thus, it is relevant to investigate the effect of MS on PD and thus, improve the understanding of the consequences of this relationship and provide information for the development of preventive strategies and treatments for MS and PD.

## METHODS

This systematic review was conducted according to the Preferred Reporting Items for Systematic Reviews and Meta-Analyses guidelines. The study protocol is registered with the International Prospective Register of Systematic Reviews (PROSPERO) (https://www.crd.york.ac.uk/prospero/; registration number: CRD42020210589).

### Eligibility criteria

#### Study design and participants

Eligibility criteria were defined based on the Population, Intervention, Comparison, Outcome and Study strategy: population, patients with PD and those with MS; comparison, between patients with and without MS; outcome, effects of MS. We included all longitudinal and cross-sectional studies on MS and PD, studies investigating the consequences of MS in patients with PD, and incidence studies investigating the association between MS and PD. Animal studies, studies on other pathologies, studies on some interventions, and revision research were excluded.

### Databases and research strategy

Two authors (APSS and JMLS) independently searched five electronic databases (Medline/PubMed, Scopus, PsycINFO, Web of Science, and Science Direct) between September and October 2020. Regarding the extraction, selection, and data collection, the two authors initially selected titles and abstracts, according to the eligibility criteria, which were later discussed to resolve any doubts or disagreements. For this stage, the descriptors MeSH “Metabolic syndrome” and “Parkinson disease” were used in all databases.

The full text of selected articles were reviewed to confirm their eligibility. Any disagreements were resolved by discussion with the third author (WMAB). The following data were collected from all articles: author names, year of publication, country, study design, study population (number, sex, and age or average age), goal, analysis methods, data collection instruments, criteria for the diagnosis of MS, biochemical parameters assessed, anthropometric parameters assessed, and general results.

The quality of all articles was assessed using the Joanna Briggs Institute (Institute JB approach available at https://joannabriggs.org/critical-appraisal-tools), an instrument to verify the reliability and relevance of selected articles. Evaluation was performed according to the study design. This step was conducted by two authors (APSS and JMLS) performed the evaluation according to the study design and any disagreements were resolved by consensus.

## RESULTS

### Selection of studies

**Figure 1:**
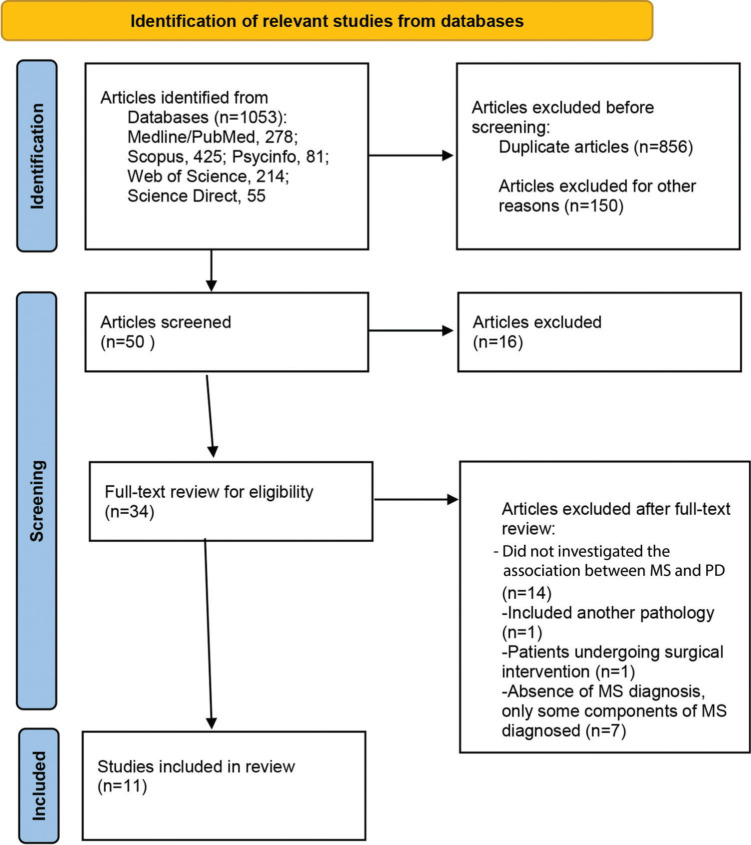
Flowchart of study selection.

The electronic search of databases yielded 1,053 articles (Medline/PubMed: 278; Scopus: 425; PsycINFO: 81; Web of Science: 214; and Science Direct: 55). After excluding 197 duplicates, the title and abstract of the remaining articles were screened based on inclusion criteria. A total of 34 articles were selected for full text review. Finally, 11 articles meeting the established eligibility criteria were included in the study (Figure 1).

### Study characteristics

This study included six cohort studies (6,7,9,15-17), four cross-sectional studies (18-21), and one case-control study (22). The locations of the included studies (N=11) were as follows: three studies, South Korea (6,7,15); two studies, the United States of America (19,20); one study, Israel (18); one study, South Africa (22); one study, China (16); one study, Canada (17); one study, Italy (21); and one study, Finland (9) (Table 1).

Overall, were evaluated data from 23,586,349 individuals (including healthy individuals with MS and PD) aged 30 years or more. In cohort studies, the follow-up period varied between 2 and 30 years. Individuals of both sexes were evaluated in all studies, except one study (22) in which only women were evaluated for methodological reasons (longer hair length in women). Three used secondary data from a national database, the National Health Insurance Service (NHIS) of South Korea (6,7,15).

The included studies used different criteria and guidelines for the diagnosis of MS, which were as follows: four studies (6,16,20,21), the National Cholesterol Education Program Adult Treatment Panel III; two studies (7,22), the Joint Interim Statement (JIS); one study, the harmonized definition of metabolic syndrome (9); and in 4 studies (15,17-19), the guideline used for the diagnosis of MS was not mentioned, but only the criteria used.

Six studies (16-18,20-22) used the same scale (the Unified Parkinson's Disease Rating Scale [UPDRS]) to assess the disease progression of PD in participants. A study (20) evaluating the progression of PD reported high UPDRS scores in individuals with MS, i.e., faster progression of PD. However, a study evaluating the association of MS with the occurrence of falls and PD progression (21) revealed that MS is associated with a reduction in the number of falls.

In the present review, the effects of MS on incidence of PD were investigated. Two studies evaluated the cognitive capacity of individuals (16,17). However, other studies evaluated this variable using common scales such as the Montreal Cognitive Assessment (17,18), the Symbol Digit Modalities Test (SDMT) (17,20), and the Mini-Mental State Examination (16,19,21).

There is little evidence in the literature regarding the effect of MS on the incidence of PD. Four studies included in this review investigated the effect of MS on the incidence of PD (6,7,9,15). Only one study (9) reported that the presence of MS indicated a 50% lower risk of developing PD. In two studies (6,7), this trend increased with the number of MS components. A study evaluating prodromal characteristics (18) did not indicate the increased risk of developing PD in individuals with MS.

### Quality evaluation of articles

No study was excluded according to the quality assessment carried out for each study design (Tables 2, 3, and 4).

## DISCUSSION

In this systematic review, we investigated the effect of MS on PD. The main finding of this study is that the incidence of PD is high among individuals with MS, confirming the research hypothesis that MS has promotive effects on PD. In addition, abdominal obesity, overweight, and obesity increase the risk of developing PD among individuals with MS (6,9,15).

Some studies, including a cohort study, investigated the incidence of PD using secondary data of 314,737 individuals aged >40 years (7). In an average 7.3-year follow-up period, 48% of the volunteers developed PD. The incidence of PD was higher in the group with MS. Similar trend was observed among patients with hypertension of both sexes. Another cohort study evaluated 6,641 individuals aged 30-79 years for a period of 30 years (9). A total of 89 individuals developed PD and overweight patients had a suggestively greater risk of PD pathology; however, presence of MS indicated a 50% lower risk of developing a neurodegenerative disease. However, although the follow-up period was long, the sample size of this study was relatively small and may have interfered with the results in terms of incidence.

In this review, a study evaluating secondary data of 17,163,560 individuals aged >40 years found that abdominal obesity increased the risk of PD (6). Moreover, with an average follow-up of 5.3 years, the study found that patients with MS are more likely to develop PD than those without MS. In addition, the study reported a positive association between PD and the number of components of MS in question.

The components of MS include increased waist circumference, a characteristic of abdominal obesity, and waist circumference can be related to dietary patterns. In this sense, a cohort study highlighted the importance of a healthy diet in association with a lower risk of PD development (23). The study evaluated 3,653 individuals of both the sexes (average age: 81.5 years) who were followed up for an average period of 6.94 years, verified the quality of the diet of the participants using a dietary screening tool, and reported 47 incident cases of PD. However, although the follow-up period was short, the number PD of cases was high. This may be because of the high average age of the participants; hence, studies investigating participants with a lower average age for a longer period if time should be conducted. In addition, the authors indicated some limitations such as the small diversity of races in the study population, lack of evaluation of energy consumption, and few food options in the questionnaire used. However, the same study meta-analyzed four more studies on the topic, including 140,617 individuals, and found that a high-quality diet or a healthy eating pattern was associated with a lower risk of developing PD.

In the search for the present review, no studies were identified associating diet, MS, and PD. However, the relationship between diet and PD can be explained by the findings of a study performed in mice (24); the study found that long-term high-fat diet suppresses receptors activated by peroxisome proliferators, increases inflammation and gliosis, and decreases dopaminergic neurons and dendritic spines in the midbrain black substance. In addition, PD mice showed neurological deficits, high anxiety, and movement disorders. Thus, it appears that the changes induced by a long-term high-fat diet interfered with both the development of morphofunctional changes in the central nervous system to facilitate the onset of PD and the existing disease.

Regarding obesity, another human study found that the availability of dopamine D2 receptors (verified by positron emission tomography) was lower in obese individuals (n=10) than in control individuals (n=10). Individuals with lower D2 values had a higher BMI. Dopamine modulates motivation and reward circuits; hence, the deficiency of this neurotransmitter in obese individuals may consolidate pathological nutrition to compensate for the alteration in the circuit (25).

A cohort study assessed the relationship between the levels of gamma-glutamyltransferase (γGT) and the risk of PD and the possible interaction between γGT and obesity or MS (15). It is already known that obesity, diabetes mellitus, and components of MS are related to the serum activity of γGT (26,27); moreover, γGT may be associated with the risk of PD development through neuroinflammation and oxidative stress, which are possible etiological factors of PD (28).

A study included in this review (15) evaluated secondary data of 6,098,405 individuals of both sexes (age: >40 years) who were followed up for an average of 6.4 years and found sex difference in the association between the serum level of γGT and the risk of PD. In men with higher serum γGT activity, the risk of developing PD was low; on the contrary, in women with higher serum γGT activity, the risk of developing PD was high regardless of age, income, BMI, smoking, alcohol consumption, and exercise level. Thus, obese women are more likely to develop PD. According to the authors, the low incidence of PD among obese men may be because of protective effect of uric acid on the development of PD (29).

In this sense, another study found a higher mean uric acid level in men with PD and MS (20). However, this study aimed to investigate the progression of PD in 1022 individuals of both sexes (individuals with MS: n=396 [mean age: 63.9 years] and individuals without MS: n=626 [mean age: 59.9 years]) using a cross-sectional design. To assess the progression of PD, we used the UPDRS, the official reference scale for assessing PD (30) and the SDMT, a cognitive test to assess the processing speed and attention of participants (31). The group with MS had higher UPDRS scores, indicating faster progression of the disease, than the group without MS; however, no difference was found in the SDMT scores between the groups. The authors suggest that treatment of MS may be a new approach to delay the progression of PD.

However, a cross-sectional study evaluating the association of MS with the occurrence of falls in 194 elderly individuals with PD (mean age: 73 years) (21) using the UPDRS found no relationship between any component of MS and the severity of PD or occurrence of falls. We believe that the small sample size affected the findings and cognitive performance because no difference was observed between individuals who reported falls and the control individuals. Similarly, a recent study found that executive dysfunction is an independent risk factor for falls in PD (32).

A cohort study investigated the effect of MS on cognition in 787 individuals with PD (16). All participants were more than 60 years old and were divided into three groups at the end of the 5-year follow-up according to cognitive function: patients with DP and normal cognitive function (PD-CN), patients with PD and mild cognitive impairment (PD-CCL), and patients with PD and dementia (DPD). The study found that the incidence of PD-CCL and PDD was higher among patients with MS than among those without MS. Moreover, patients who received treatment for MS had a lower risk of PPD. Patients with PD-CCL and those with PPD had higher levels of hypertension, glucose, and triglycerides than those with PD-CN. In addition, high triglyceride levels were associated with executive function, language, memory, and visuospatial function in patients with PPD. Thus, the authors suggested that treating MS can be useful in controlling cognitive impairment in PD, confirming the hypothesis that MS affects PD progression.

## CONCLUSIONS

Systematic review of studies revealed that MS contributes considerably to the increase in the incidence of PD. In addition, obesity alone can increase the probability of developing neurodegenerative diseases. However, despite few studies on the relationship between MS and PD, changes in cognitive function and the most rapid progression of PD has been documented in patients with MS, using methods frequently used in research. Hence, the scientific community should focus on the relationship between MS and PD because its understanding can promote advances in the control and prevention of both MS and PD.

## AUTHOR CONTRIBUTIONS

Souza APS, Barros WMA and Silva JML contributed to research conception, data collection, interpretation of results and critical review of the manuscript. Silva MRM, Silva ABJ, Fernandes MSS and Santos MERA contributed to data analysis and interpretation, and manuscript drafting. Silva ML, Carmo TS, Silva RKP and Silva KG contributed to manuscript drafting and critical review. Souza SL and Souza VON reviewed the manuscript.

## Figures and Tables

**Table 1:** Main characteristics of the included studies.

Study, year, Country, Study design	Sample (number, sex, age or mean age)	Objective	Methods	Data collection instruments	Diagnosis of MS and related component	Results: biochemical parameters	Results: anthropometric variables	Main Results
**Roh et al. (7)** **South Korea** **Cohort study**	N= 314.737 56.1% M 85,530 individuals with MS and 85,530 individuals without MS ↑ 40 years old	To investigate the effect of MS on the incidence of PD	Analysis of data from the National health service database. (NHIS-HealS) (2002-2015)	Secondary data	Joint Interim Statement (JIS)	↓ HDL ↑ PD incidence ↓ Correlation between HDL and PD risk observed in men ↑ TG associated with the incidence of PD in individuals in general ↑ Fasting glucose associated with ↑ PD incidence in individuals in general	↑ WC was not associated with the incidence of PD in individuals in general.	Average follow-up period: 7.23 years 819 (0.48%) individuals developed PD ↑ Incidence among individuals with MS ↑ Hypertensive 1.34 incidence of PD in both sexes ↑ Incidence of PD increased with the number of components of MS
**Thaler et al. (18)** **Israel** **Cross-sectional study**	N=562 M and F 104 individuals with idiopathic PD, 40 individuals with LRRK2 -PD, 70 individuals with GBA-PD, 196 healthy carriers, 55 individuals with LRRK2 -PNM and 97 individuals with GBA -PNM	To evaluate the effect of MS on the phenotype of LRRK2 and PD GBA and on the prevalence of prodromal characteristics among individuals at risk for PD	Genetic tests for the G2019S mutation in the LRRK2 gene and for the AJ GBA mutations; laboratory tests; Information; cognitive, motor, olfactory and affective functions.	MDS-UPDRS; MoCA, BDI; NMSQ; SCOPA-AUT; RBDO; UPSIT. Demographic data; PAN; Blood analysis of HbA1c, TG and HDL levels.	Change in 3 of the 5 components: fasting glucose levels, BP, BMI, HDL, and TG.	*LRRK2* -PD ↑ TG levels and pre-diabetes rates. *LRRK2 -PNM TG levels* PNM with probability rates for prodromal PD. ↑ 50% had frequencies of hypertriglyceridemia and pre-diabetes. Pre-diabetes associated with age and UPDRS-III scores accounting for 38.8% of the variance. The probability ratio for conversion to PD in 10 (3%) individuals was >80% (non-carriers: 4 individuals, GBA-PNM: 3 individuals, and LRRK2-NMC: 3 individuals).		Groups with PD and PNM did not differ in the number of metabolic components 70 PD patients (32.7%) with MS. This group was older and had a longer diagnosis time. 94 (27.2%) PNM with MS. Hypertension was associated with age and sex M was responsible for 35.7% of the variance. Individuals with MS did not have a high risk of developing PD.
**van den Heuvel et al. (22)** **South Africa** **/>Case-control study**	56 F (25 pcts F with PD and 31 controls) aged 45 to 78 years	To examine the association of PD diagnosis, clinical characteristics, and risk of PD-CVD (as defined by the MS) with the levels of GC (cortisol and cortisone) in hair.	Analysis of hair samples, representing a 3-month retrospective window of GC levels using tandem mass chromatography liquid chromatography.	Glycemia, TG, HDL, HbA1c; LDL, total blood cholesterol. Sociodemographic data, MINI, UPDRS, H&I, NMSS; PSS-10; WHO STEPS; GPAQ.	Joint Interim Statement(JIS)	↑ Capillary cortisone in PD patients than in controls. Levels of cortisone in hair in unadjusted analysis were associated with the number of criteria for MS, use of antihypertensive medication, WHR, known CVD and use of aspirin, and a tendency to demonstrate significance for HDL-C.		Prevalence of MS in Pcts with PD was 56.0% and that in control individuals was 25.8%. Non-motor symptoms of PD (mood anhedonia and anxiety) were associated with capillary cortisone levels. No significant interactions were found between PD and MS based on the GC levels in hair.
**Yoo et al. (15)** **South Korea** **Cohort study**	N=6,098,405 F and M >40 years old	To assess the association between the serum GGT level and PD risk. Analyze the possible interaction between the GGT level and obesity or MS	National Health Insurance Service (NHIS) database	Secondary data	Change in 3 of the 5 components or use of antihypertensive, hypoglycemic, or hypolipemic medication.	-	Factors associated with the serum GGT level were age, low income, BMI, current smoking, light to moderate alcohol consumption, exercise, DM, hypertension, dyslipidemia, chronic kidney disease, and MS	Average follow-up period was 6.4 years. DP developed in 20,895 (0.34%) individuals (M: 9,512 and F: 11,383) ↓ PD incidence was low in ↑ M, whereas ↑ high in F according to GGT activity ↑ Obesity and MS are the risk factors for PD in both sexes. A sub additive interaction was found between the serum GGT level and obesity in women.
**Nam et al. (6)** **South Korea** **Cohort study**	N=17,163,560 age ≥40 years Individuals with MS: 58.1 years Individuals without MS: 51.9 years	To investigate the association of MS and its components with the development of PD in the South Korean population using large cohort data.	Analysis of NHIS data from January 1, 2009, to December 31, 2012 and follow-up of pcts up to 31 December 2015.	Secondary data	National Cholesterol Education Program Adult Treatment Panel III.	Pcts with 3 MS components at ↑ 31% PD risk, and those with all 5 components at ↑ 66% risk compared with those without any component	MS group had higher mean BMI, WC, BP, glucose level, serum total cholesterol level, TG level, and LDL-C level. Individuals with abdominal obesity or hypertriglyceridemia had approximately 13% PD risk.	Average follow-up period was 5.3 years. 44,205 incident cases of PD. ↑ Individuals with MS (n=5,848,508) had a higher risk of PD than individuals without MS (n=11,315,052). Each component of MS was positively associated with the risk of PD. Positive correlation was found between the incidence of PD and the number of components of MS.
**Peng et al. (16)** **China** **Cohort study**	N=787 60 years. Individuals with PD divided into three groups: PD-CN, PD-CCL and PDD. 249 individuals with PD and MS at the beginning of the study divided into 3 subgroups according to the treatment of each component of MS: untreated pcts, pcts who received treatment for some components, and pcts who received treatments for all components.	To investigate the effect of MS on cognitive impairment in PD.	Physical and neurological examinations, blood analysis and neuroimaging. MS incidence assessed based on medical records or doctors' assessment. Annual monitoring for 5 years.	UPDRS, MMSE, RAVLT, Doppler ultrasonography, computed tomography angiography, magnetic resonance imaging	National Cholesterol Education Program's Adult Treatment Panel III.	↑ Individuals with PD-CCL and those with PDD had high levels of hypertension, glucose, and ↑ hypertriglyceridemia than individuals with PD-CN. Associations between BP, glucose, TG, and PDD. BP and glucose levels were associated with the main cognitive domains; TG level was associated with executive function, language, memory, and visuospatial function; HDL-C level was associated with memory and visuospatial functions.	Individuals with PD-CCL and those with PDD were significantly older and had higher BMI, UPDRS 3 scores, H&Y stage, systolic and diastolic BP, glucose level, total cholesterol level, and TG level as well as longer duration of PD. Still had lower education level (<6 years), lacunar infarction, white matter lesions, and MS than those with PD-CN	At 5 years, 255 (32.4%) individuals were diagnosed with PD-CCL and 105 (13.3%) individuals were diagnosed with PDD. MS was associated with PD-CCL. MS was associated with the main cognitive domains in Pcts with PDD. Incidence of PD-CCL and PDD was higher in individuals with MS than in those without MS Pcts treated for some or all of the major components of MS had a ↓ lower risk of PDD than patients with untreated MS.
**Oxenkrug et al. (19)** **United States of America (USA)** **Cross-sectional study**	7 F pcts with PD and 11 M pcts with PD (age range: 50 to 74 years). 12 F pcts with probable AD and 8 M pcts with probable AD (age range: 60 to 75 years). 24 healthy individuals matched for age and gender (12 F and 12 M).	To compare peripheral quinurenines in AD and PD with an emphasis on quinurenines associated with MS (i.e., KYNA, ANA, 3-HK, and XA)	Analysis of blood samples collected after overnight fasting.	Analysis of Trp, Kyn, ANA, KYNA, and 3-HK using high performance liquid chromatography coupled with mass spectrometry, MMSE		N/S differences in plasma concentrations of Trp, Kyn, and all Kyn metabolites were found in untreated patients and patients treated with L-DOPA.		In Pcts with PD, ↓ Trp concentrations and the reason Kyn: Trp, Kyn, ANA and KYNA ↑ than in the control. Concentrations of 3-HK in Pcts with PD were below the sensitivity limit of the method. In Pcts with AD, the serum ANA concentrations were approximately ↑ 3 times higher and KYNA concentrations were approximately ↓ 40% more than those in control individuals.
**Leehey et al. (20)** **USA** **Cross-sectional study**	N=1022 M and F 2 groups: Individual with MS (N=396, mean age 63.9 years) over the 3 years of the study and individuals without evidence of MS (N=626, mean age 59.9 years) over the 3 years.	To compare the progression of PD between individuals with MS during the first 3 years of the trial and individuals with no evidence of MS.	Secondary analysis of patient data from the NET-DP LS study 1. The changes in the UPDRS and SDMT scores from randomization to 3 years were compared.	UPDRS, SDMT	National Cholesterol Education Program Adult Treatment Panel III (modified).	-	-	Pcts with older MS: propensity to be of the sex M (75.3% *versus* 57.0%), and ↑ an average level of uric acid. In Pcts with MS there was an ↑ additional 0.6- (0.2) units per year in the total UPDRS and ↑ 0.5- (0.2) units in the motor UPDRS scores compared with participants without MS. N/S changes in SDMT scores.
**Doiron et al. (17)** **Canada** **Cohort study**	367 pcts (≥50 years old) with PD at baseline and 310 pcts at 24-month follow-up.	To investigate hypertension, dyslipidemia, DM, and BMI as possible risk factors for cognitive impairment in a large cohort of untreated patients with PD and without dementia.	Clinical and neurological examinations, biological sampling, neuropsychological assessments, and neuroimaging. Participants reassessed approximately after 24 months.	UPDRS, GDS, Girl, LNS, SDMT, HVLT-R, BJLO, total blood cholesterol, LDL, HDL, and TG.	Hypertension, DM, Obesity	HDL was correlated with BJLO performance at baseline	BMI was associated only with the verbal fluency Z score at 24 months.	Hypertension was the most prevalent comorbidity in 35.4% of the individuals at baseline and 41.1% of the individuals at follow-up. Time of hypertension and pulse pressure were associated with worse cognitive outcomes. Longer history of hypertension and ↑ higher pulse pressure were associated with impaired verbal episodic memory and semantic verbal fluency. Other vascular risk factors were not associated with cognitive outcomes.
**Laudisio et al. (21)** **Italy** **Cross-sectional study**	N=194 Elderly individuals with PD (IM 73 years old) treated at a geriatric hospital at the Catholic University of Rome.	To evaluate the association between MS and occurrence of falls in patients with PD.	Recorded the history and number of falls in the last year.	ADL; IADLs; GDS; MMSE, UPDRS, MNA, dual energy X-rays Absorptiometry, Portable dynamometer, Tinetti, 24-h BP recording.	National Cholesterol Education Program Adult Treatment Panel III.	N/S associations were noted between the occurrence of falls and any of the components of SM.	-	91 (47%) participants reported falls. MS was diagnosed in 44 (23%) participants. Severity of PD according to the UPDRS did not differ significantly according to the diagnosis of MS. SM was associated with less falls.
**Sääksjärvi et al. (9)** **Finland** **Cohort study**	6641 aged 30-79 years and without PD at baseline (1978-1980)	To investigate whether MS or its components, or serum total cholesterol, can predict the incidence of PD in a prospective cohort.	Based on the Mini-Finland Health Survey conducted from 1978 to 1980. Anthropometric, biochemical blood analyses were performed.	HDL, TG, Glycemia, and total cholesterol in the blood. 25-hydroxy vit D serum by radioimmunoassay	Harmonized definition of the metabolic syndrome	↑ Serum TG and fasting plasma glucose predicted ↓ risk of PD, even after excluding the first 10 years of follow-up. PA, HDL, or total cholesterol do not predict PD risk.	↑ Risk suggestive of PD observed in overweight individuals	During 30-year follow-up (1978-2007), 89 incident cases of PD were recorded. MS predicted the risk of PD up to 50%.

Abbreviations: 3-HK, 3-hydroxyquinurenine; AD, Alzheimer’s disease; ADLs, Katz’s activities of daily living; ANA, anthranilic acid; BDI, Beck Depression Inventory; BJLO, Benton Judgment of Line Orientation; BMI, body mass index; BP, blood pressure; CC, waist circumference; CVD, cardiovascular disease; F, female; GBA, glucocerebrosidase genes; GC, glucocorticoid; GDS, Geriatric Depression Scale; GGT, serum gamma-glutamyltransferase; GPAQ, global physical activity questionnaire; HbA1c, Glycated hemoglobin; HDL, high-density lipoprotein; H HVLT-R, Hopkins Verbal Learning Test Revised; IADL, Lawton and Brody scale for instrumental activities of daily living; KYNA, quinurenic acid; LDL, low-density lipoprotein; LNS, Letter–Number Sequencing; LRRK2-repeat of leucine-rich kinase 2; M, male; MINI, Mini International Neuropsychiatric Interview; MMSE, Mini-Mental State Examination; MNA, Mini Nutritional Assessment; MoCa, Montreal Cognitive Assessment; MS, Metabolic syndrome; NET-PD LS 1, National Institute of Neurological Diseases and Stroke Exploratory Trials in the Long Term Study 1 of PD; NHIS-HEALS National Health Insurance Service-National Health Screening Cohort; NMSQ, Non-Motor Symptoms Questionnaire; NMSS, Non-Motor Symptoms Scale; N/S, not significant; Pcts-patients; PD, Parkinson’s disease; PD-CN, Parkinson’s disease with normal cognitive function; PDD, Parkinson’s disease and dementia; PD-CCL, mild cognitive impairment in PD; PNM, Carrier not manifest; PSS10, Perceived Stress Scale; RAVLT, Rey Auditory Verbal Learning Test; RBDO-REM, Sleep Behavior Disorder Questionnaire; SCOPA-AUT, Scale of Autonomic Function in PD; SDMT, Symbol Digit Modalities Test; TG, triglycerides; Trp, Tryptophan; UPDRS, Unified Parkinson’s Disease Rating Scale; UPSIT, University of Pennsylvania Smell Identification Test; WHO STEPS, WHO STEPwise approach; WHR, waist to hip ratio; XA, xanthurenic acid.

**Table 2:** Results of the critical assessment of the included studies (Cohort) using the Joanna Briggs Institute approach.

Studies	Q1	Q2	Q3	Q4	Q5	Q6	Q7	Q8	Q9	Q10	Q11
**(7)**	Y	Y	Y	Y	Y	Y	Y	Y	U	U	Y
**(15)**	Y	Y	Y	Y	Y	Y	Y	Y	U	U	Y
**(6)**	Y	Y	Y	Y	Y	Y	Y	Y	U	U	Y
**(16)**	Y	Y	Y	Y	Y	Y	Y	Y	Y	Y	Y
**(17)**	Y	Y	Y	Y	Y	Y	Y	Y	Y	U	Y
**(9)**	Y	Y	Y	Y	U	Y	Y	Y	Y	Y	Y

N, no; NA, not applicable; U: unclear; Y, yes. Q1: Were the two groups similar and recruited from the same population? Q2: Were the exposures measured similarly to assign individuals to both the exposed and unexposed groups? Q3: Was the exposure measured using a valid and reliable method? Q4: Were confounding factors identified? Q5: Were strategies to overcome the confounding factors stated? Q6: Were the groups/participants free of the outcome at the start of the study (or at the time of exposure)? Q7: Were the outcomes measured using a valid and reliable method? Q8: Was the follow-up period reported and sufficiently long for outcomes to occur? Q9: Was follow-up complete, and if not, were the reasons for loss to follow-up described and explored? Q10: Were strategies to address incomplete follow-up used? Q11: Was an appropriate statistical analysis method used?

**Table 3:** Results of the critical assessment of the included studies (Cross-sectional) using the Joanna Briggs Institute approach.

Studies	Q1	Q2	Q3	Q4	Q5	Q6	Q7	Q8
**(18)**	Y	Y	Y	Y	Y	Y	Y	Y
**(19)**	U	U	Y	Y	U	U	U	U
**(20)**	Y	Y	Y	Y	Y	Y	Y	Y
**(21)**	Y	Y	Y	Y	Y	Y	Y	Y

N, no; U, unclear; Y, yes. Q1: Were the inclusion criteria clearly defined? Q2: Were the study participants and context described in detail? Q3: Was exposure measured using a valid and reliable method? Q4: Were objective and standard criteria used to measure the condition? Q5: Were the confounding factors identified? Q6: Were strategies to overcome confounding factors used? Q7: Were the results measured using a valid and reliable method? Q8: Was an appropriate statistical analysis method used?

**Table 4:** Results of the critical assessment of included studies (case-control) using the Joanna Briggs Institute approach.

Studies	Q1	Q2	Q3	Q4	Q5	Q6	Q7	Q8	Q9	Q10
**(22)**	Y	Y	Y	Y	Y	Y	Y	Y	Y	Y

N, no, NA, not applicable; U, unclear, Y: Yes. **Q1:** Were the groups comparable, except for the presence of disease in patients and absence of disease in control individuals? **Q2**: Were patients and control individuals matched appropriately? **Q3:** Were the same criteria used for the identification of patients and control individuals? **Q4:** Was exposure measured using a standard, valid, and reliable method? **Q5:** Was the same method used to measure the exposure in both patients and control individuals? **Q6:** Were confounding factors identified? **Q7:** Were strategies to overcome the confounding factors stated? **Q8:** Were outcomes assessed using a standard, valid, and reliable method in patients and control individuals? **Q9:** Was the exposure period sufficiently long to be meaningful? **Q10:** Was an appropriate statistical analysis method used?
